# Activity-Guided Fractionation of Red Fruit Extracts for the Identification of Compounds Influencing Glucose Metabolism

**DOI:** 10.3390/nu11051166

**Published:** 2019-05-24

**Authors:** Johanna Josefine Ostberg-Potthoff, Kirsten Berger, Elke Richling, Peter Winterhalter

**Affiliations:** 1 Institut für Lebensmittelchemie, Technische Universität Braunschweig, Schleinitzstraße 20, D-38106 Braunschweig, Germany; j.ostberg@tu-braunschweig.de; 2 Department of Chemistry, Division of Food Chemistry and Toxicology, Technische Universität Kaiserslautern, Erwin-Schrödinger-Straße 52, D-67663 Kaiserslautern, Germany; berger@chemie.uni-kl.de (K.B.); richling@chemie.uni-kl.de (E.R.)

**Keywords:** aronia, pomegranate, red grape, polyphenols, HPLC-PDA-MS/MS, α-amylase, α-glucosidase, high-performance countercurrent chromatography (HPCCC)

## Abstract

An activity-guided search for compounds influencing glucose metabolism in extracts from aronia (*Aronia melanocarpa*, A.), pomegranate (*Punica granatum L.*, P.), and red grape (*Vitis vinifera, RG*) was carried out. The three extracts were fractionated by means of membrane chromatography to separate the anthocyanins from other noncolored phenolic compounds (copigments). In addition, precipitation with hexane was performed to isolate the polymers (PF). The anthocyanin and copigment fractions (AF, CF) of aronia, pomegranate, and red grape were furthermore fractionated with high-performance countercurrent chromatography (HPCCC) and the subfractions were characterized by HPLC-PDA-MS/MS analyses. Each of the (sub-)fractions was examined by in vitro-tests, i.e., the inhibition of the activity of α-amylase and α-glucosidase. On the basis of this screening, several potent inhibitors of the two enzymes could be identified, which included flavonols (e.g., quercetin), ellagitannins (e.g., pedunculagin), and anthocyanins (e.g., delphinidin-3-glucoside and petunidin-3-glucoside). In the α-glucosidase assay all of the examined fractions and subfractions of the fruit extracts were more active than the positive control acarbose.

## 1. Introduction

Hyperglycemia is an increasing problem all over the world and many studies point out the risks between hyperglycemia and the development of diabetes, cardiovascular diseases and cancer [1,2]. Many polyphenols are discussed in the context of reducing blood glucose levels. High concentrations of polyphenolic compounds, such as anthocyanins, have shown a potential for reducing hyperglycemia [3,4,5]. Furthermore, polyphenols inhibited enzymes involved in the glucose metabolism, for example α-glucosidase and α-amylase [6]. Polyphenols, especially flavonoids, interact over the H-bond between the enzyme and hydroxyl groups and π–π interactions stabilized the inhibition of the carbohydrate digestion enzymes at the catalytic centers [3]. The inhibition potential of polyphenol rich extracts from aronia (*Aronia melanocarpa*), pomegranate (*Punica granatum L.*), and red grape (*Vitis vinifera*) were tested in vitro against α-glucosidase and α-amylase. Active compounds, which may affect in vivo the blood metabolism and decrease the blood glucose level, were attempted to be identified using a structure–activity guided fractionation approach. For the separation of anthocyanins and copigments (i.e., colorless phenolic compounds), a cellulose membrane with sulfonic acid groups on the surface can be used [7,8]. The positively charged flavylium cations of the acidified anthocyanins are retarded by the sulfonic acid groups. Copigments are not adsorbed and elute first, separated from the anthocyanins. In addition, solvent precipitation with hexane yields a fraction of polymeric phenolic compounds [9,10]. For the (sub-)fractionation of the anthocyan and copigment fractions, high-performance countercurrent chromatography (HPCCC) is applied. HPCCC is a fast and gentle method for the preparative separation of natural compounds [11,12,13]. Separation with HPCCC yields in many cases pure compounds, which subsequently can be tested with regard to their inhibition potential. In the present study we describe the successful identification of compounds from aronia, pomegranate, and red grape which influence the glucose metabolism in vitro.

## 2. Materials and Methods 

### 2.1. Chemicals

Double deionized water was prepared using Nanopure^®^ resin (Werner, Leverkusen, Germany). Acetonitrile (HPLC and LC-MS grade) was obtained from Honeywell Specialty (Seelze, Germany) and formic acid (LC-MS grade) and *n*-hexane (HPLC grade) were supplied by Fisher Scientific (Loghborough, UK). Formic acid (analytical grade), sodium hydroxide (≥99%), *n-*butanol (HPLC grade), and sodium chloride (≥99%) were obtained from Carl Roth (Karlsruhe, Germany). Acetic acid (≥99.8%), ethanol (≥99.8%), trifluoroacetic acid (99%), 2,2′-azino-bis(3-ethylbenzothiazoline-6-sulfonic acid)diammonium salt (ABTS), (±)-6-hydroxy-2,5,7,8-tetramethylchromane-2-carboxylic acid (Trolox, 97%) and Amberlite^®^ XAD-7 HP were obtained from Sigma-Aldrich (Steinheim, Germany). Aqueous hydrochloric acid (37%) was ordered from VWR Int. S.A.S. (Darmstadt, Germany). Folin-Ciocalteu’s phenol reagent was obtained from Merck (Darmstadt, Germany). Gallic acid monohydrate (≥98%), *tert-*butyl methyl ether p.a. grade (≥99.5%), and sodium carbonate (≥99%) were obtained from Fluka (Buchs, Switzerland). Potassium peroxodisulfate p.a. was obtained from Riedel-de Haën (Seelze, Germany). 2-Chloro-4-nitrophenol-α-d-maltotrioside, 4-nitrophenyl-α-d-glucopyranoside, α-amylase from porcine pancreas, α-glucosidase from *Saccharomyces cerevisiae* were purchased from Sigma-Aldrich (Taufkirchen, Germany). Acarbose was obtained as Glucobay^®^ (50 mg acarbose/tablet) from Bayer Pharmaceuticals (Leverkusen, Germany). 

### 2.2. Samples

The aronia NFC (NFC = not from concentrate) (*Aronia melanocarpa*), pomegranate NFC (*Punica granatum L.*) were from Haus Rabenhorst O. Lauffs GmbH & Co. KG (Unkel, Germany) and the red grape JC (JC = juice concentrate) (*Vitis vinifera*) was from Eckes-Granini Deutschland GmbH (Nieder-Olm, Germany). 

### 2.3. Membrane Chromatography (MC)

In order to separate the anthocyanins from the copigments, a cellulose membrane was used. This application was established by Juadjur [7]. A membrane adsorber type Sartobind S IEX 150 mL, a Sartopore 2300 filter capsule and a Tandem 1082 peristaltic pump from Sartorius (Göttingen, Germany) with a flow rate of 100 mL/min was used. Amberlite^®^ XAD-7 red juice extracts (~5 g) were dissolved in 1 L ethanol/acetic acid (19:1; v/v) and passed through a MN 615 ¼ filter (Machery-Nagel, Düren, Germany). Prior to the separation the membrane was regenerated with 2 L NaOH (1 N) and equilibrated with 2 L HCl (0.01 N) and 1.5 L ethanol/acetic acid (19:1; v/v). The membrane adsorber was then loaded with the dissolved extract (ethanol/acetic acid (19:1; v/v)). Copigments were eluted with 1 L of a mixture of ethanol/acetic acid (19:1; v/v). The solvents of the copigment fraction (CF) were removed in vacuo and the residue was freeze-dried. The retained anthocyanins were eluted with 1 L of an aqueous NaCl solution (1 N)/ethanol (1:1; v/v). For stabilization, the anthocyanin fraction (AF) was acidified with acetic acid and concentrated in vacuo and the residue was freeze-dried. For removal of NaCl from the anthocyanin fraction, an Amberlite^®^ XAD-7 column was used. The column was washed with 1 L ethanol and equilibrated with 1 L water. The anthocyanin fraction was dissolved in water and applied onto the XAD-7 column. After the column was washed with water, the anthocyanin fraction was eluted with 1 L ethanol/acetic acid (19:1; v/v). Solvents were evaporated in vacuo and the residue was freeze-dried.

### 2.4. Polymer Precipitation (PP) Using Hexane

Hexane precipitation of polymeric phenolic compounds was used to separate the polymers from the monomers. The Amberlite^®^ XAD-7 extract (~5 g) was dissolved in 1 L ethanol and was stirred for 1 h. Then *n-*hexane was added at a rate of 10 mL/min. The precipitate was removed by filtration, dissolved in water, and freeze-dried.

### 2.5. Countercurrent Chromatography (CCC)

A high-performance CCC “Spectrum” (Dynamic Extractions Ltd., Tredegar, UK) was equipped with two columns (PFE tubing i.D. 1.6 mm, total coil volume 125.5 mL). A RC6 CS thermostat from Lauda R. Wobser (Lauda-Königshofen, Germany) set the temperature to 30 °C; flow rate was 3 mL/min (RT.ing MP-2001 HPLC-pump, Potsdam, Germany). Elution was monitored with a Well-Chrom Spectro-Photometer K-2500 detector from Knauer (Berlin, Germany) at a wavelength of λ 520 nm. Fractions of three mL were collected with a Pharmacia LKB Super Frac (Bromma, Sweden). Eurochrom 2000 (Windows version) software from Knauer (Berlin, Germany) was used. The separations were run at a revolution speed of 1600 rpm and the elution mode was head to tail, the upper phase being used as the stationary phase. Freeze-dried copigment or anthocyanin fractions were dissolved in a 1:1 (v/v) mixture of lower and upper phase and injected into the system by loop injection (5 mL). Sample amount varied from 400 to 1000 mg. Stationary phase retention was in a range of 61 to 78%. After fractionation, solvents were evaporated in vacuo and the residue was freeze-dried. Solvent system for the anthocyanin separation of aronia NFC and red grape JC and copigment fraction of pomegranate NFC consisted of *tert-*butyl methyl ether/*n*-butanol/acetonitrile/water (2:2:1:5; v/v/v/v) acidified with trifluoracetic acid [14]. The same solvent system without trifluoracetic acid was used for the copigment fraction of aronia NFC. For the separation of pomegranate NFC anthocyanin fraction, a mixture of *tert-*butyl methyl ether/*n*-butanol/acetonitrile/water (1:3:1:5; v/v/v/v) acidified with trifluoracetic acid was applied. In the case of red grape JC copigment fraction, a mixture of *tert-*butyl methyl ether/acetonitrile/water (2:2:3; v/v/v) was used.

### 2.6. HPLC-PDA-ESI-MS/MS

The HPLC system (1100/1200 series, Agilent, Waldbronn, Germany) consisted of a binary pump (G1312A), an autosampler (G1329B), and a DAD-detector (G1316A). It was coupled to a HCT Ultra Ion Trap mass spectrometer (Bruker Daltonics, Bremen, Germany) with an electrospray ionization source (ESI). The anthocyanins and copigments were separated on a Luna C18(2) 3 μ column (150 × 2.0 mm, Phenomenex (Torrance, CA, USA)) using water/acetonitrile/formic acid (95/3/2; v/v/v) (eluent A) and water/acetonitrile/formic acid (48/50/2; v/v/v) (eluent B) at a flow rate of 200 μL/min. Gradient elution was performed, starting with 6% eluent B and rising to 35% over 30 min. The level of eluent B was then set to 40% until minute 35, and then 90% until minute 45. This level was maintained for 5 minutes before being reduced to 30% until minute 55. Finally, the initial conditions (6% eluent B) were restored until minute 70. The ESI source was operated in positive mode (anthocyanins), negative mode (copigments) and alternating mode (+/−3000 V), using nitrogen as the nebulizer (50 psi) and drying gas (10 L/min, 365 °C). The sample extracts were dissolved in 2 mL of eluent A. Aliquots of 5 μL of each sample were analyzed by the HPLC-PDA-ESI-MS/MS method described above using the Bruker Hystar 3.2, Bruker ESICompass 1.3 for HCT/Esquire, and Data Analysis Version 3.0 software packages (Bruker Daltonics, Bremen, Germany).

### 2.7. HPLC-PDA

The HPLC system was from Jasco (Groß-Umstadt, Germany) consisting of an Intelligent HPLC pump PU-2080 Plus, 3-Line-Degasser DG-2080-50, Ternary Gradient Unit LG-2080-02, Intelligent Sampler AS-2057 Plus, Multiwavelength Detector MD-2010 Plus, Intelligent Column Oven CO-2067 Plus, a LC-NetII/ADC transmitter and Chrompass Chromatography Data System V. 1.8. software. The separation was performed on a SIELC Primesep B2 5 µ (150*2.1 mm) guard column (Wheeling, USA). Analyses were performed using eluent A with 5% formic acid and eluent B acetonitrile at flow rates of 200 and 400 µL/min. Gradient elution was performed, starting with 0% eluent B for 20 minutes rising to 15% over minute 60. The level of eluent B was then set to 50% until minute 70. The flow rate changed from minute 65 from 200 to 400 µL/min, with eluent B rising to 100% over 5 min. At minute 80 the flow rate was set to 200 µL/min. The 100% level B was maintained for 5 min before being reduced to initial conditions (0% B) until minute 85. Finally, the initial conditions were restored until minute 100. Anthocyanins were detected at λ 520 nm and copigments at λ 280 and 320 nm.

### 2.8. In Vitro α-Amylase Inhibition Study

The α-amylase inhibition assay is based on a previously described spectrophotometric method [15]. Acarbose is used as the reference compound. Briefly, 20 μL aliquots of each of the sample, the positive control, and the negative control were mixed with 70 μL of 40 mM phosphate buffered saline (PBS) at pH 6.9 containing 0.5 mg/mL of porcine pancreatic α-amylase and incubated in a 96-well microplate at 37 °C for 10 min. After preincubation, 100 μL of substrate solution (40 mM PBS with 4 mM 2-chloro-4-nitrophenyl-α-d-maltotrioside) is added, and the resulting mixtures are incubated at 37 °C for 8 min. Their absorbance is then measured at λ 405 nm using a microplate reader (Biotek, Bad Friedrichshall, Germany), and the percentage of inhibition is calculated using the expression:

(1) $$\%-Inhibition=\left(\frac{absorbance_{control}-absorbance_{sample}}{absorbance_{control}}\right)*100$$

The IC_50_ value is calculated by linear regression and expressed in mg/mL.

### 2.9. In Vitro α-Glucosidase Inhibition Study

The α-glucosidase inhibition assay uses 4-nitrophenyl-α-d-glucopyranoside as substrate [16]. Twenty microliter aliquots of the sample and negative and positive control solutions were each mixed with 70 μL enzyme solution (1 U/mL) in 0.1 M PBS (pH 6.8). After incubation at 25 °C for 10 min, 100 μL of 4 mM pNPG solution in PBS is added and incubated in a 96-well microplate for 5 min at 25 °C. The absorbance is measured at λ 405 nm using a microplate reader (Biotek, Bad Friedrichshall, Germany). Percentage inhibition and IC_50_ values were calculated as described above, and the results are expressed in µg/mL. 

## 3. Results

### 3.1. Separation of Phenolic Compounds into Three Fractions (Anthocyanins, Copigments and Polymers)

Amberlite XAD-7 extracts of aronia NFC (A, *Aronia melanocarpa*), pomegranate NFC (P, *Punica granatum* L.), and red grape JC (RG, *Vitis vinifera*) were prepared as described in the literature [17]. For the further separation of anthocyanins and copigments membrane chromatography [7] has been applied. A third fraction (polymeric phenols) was obtained by hexane precipitation [9].

#### 3.1.1. Separation with Membrane Chromatography

**Figure 1:**
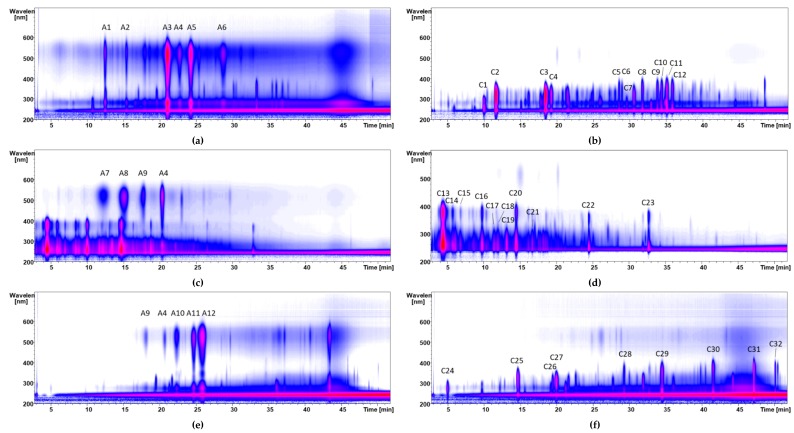
Isocontour plots of the high-performance liquid chromatography electrospray ionization tandem mass spectrometry (HPLC-ESI-MS/MS) chromatograms of the anthocyanin fractions of aronia not from concentrate (NFC) (**a**), pomegranate NFC (**c**), and red grape JC (**e**), as well as the copigment fractions of aronia NFC (**b**), pomegranate NFC (**d**), and red grape JC (**f**) monitored from λ 200 to 600 nm. Peak identification is given in Table 1.

Our membrane chromatography method [7] enabled the separation of anthocyanins and copigments. Anthocyanins are first acidified and converted into flavylium cations. The charged anthocyanin molecules are then retarded at the sulfonic acid groups of the membrane adsorber. Neutral copigments are not retained. They elute immediately separated from the anthocyanins. One M NaCl/ethanol (1:1; v/v) solution is used for the subsequent desorption of the anthocyanins. Desalination of the anthocyanin fraction is achieved by Amberlite^®^ XAD-7 column chromatography. The purity of the fractions was monitored by high-performance liquid chromatography electrospray ionization tandem mass spectrometry (HPLC-ESI-MS/MS) analyses. Thirty-seven percent of anthocyan fraction and 54% of copigment fraction were obtained from the aronia XAD-7 extract. Nine percent of the sample (polymeric compounds) was retained by the cellulose membrane, which could be recovered by a rinsing step with 1 N aqueous NaOH. Pomegranate contains few anthocyanins, but is abundant in tannins. The high content of the tannins influenced the separation of the anthocyanins by membrane chromatography, i.e., more solvent for the complete elution of the copigments had to be used. After the separation, 12% of an anthocyanin and 66% of a copigment fraction were obtained. The red grape JC extract contained 24% of anthocyanins and 49% of copigments. The remaining 27% were polymeric components. Figure 1 shows the isocontour plots of the anthocyanin and copigment fractions of aronia NFC, pomegranate NFC and red grape juice. LC-MS data on molecular ions and fragment ions of 12 anthocyanins and 32 copigments is given in Table 1. The identification of polyphenolic compounds is based on comparison of the MS and MS/MS data, retention times, and UV spectra with authentic standards as well as published data.

In the case of the aronia NFC sample, cyanidin-3-galactoside was detected as major anthocyanin together with two cyanidin derivatives with *m/z* 737 and 707. The first cyanidin derivative was identified as cyanidin-3,5-hexoside-(epi)catechin und the second as cyanidin-3-pentoside-(epi)catechin [18]. In addition, cyanidin-3-glucoside with the [M-H]^+^ molecular ion at *m/z* 449, cyanidin-3-arabinoside with the [M-H]^+^ molecular ion at *m/z* 419, and cyanidin-3-xyloside [M-H]^+^ molecular ion at *m/z* 419 were identified as described in the literature [18,19,20]. The main copigment of aronia NFC was 3-caffeoylquinic acid (neochlorogenic acid, *m/z* 353) together with 5-caffeoylquinic acid (chlorogenic acid), and 4-caffeoylquinic acid (cryptochlorogenic acid). 

Four anthocyanins and eleven copigments were identified in the pomegranate NFC sample. In this case, mainly diglycosidically bound anthocyanins were detected, such as cyanidin-3,5-diglucoside or delphinidin-3,5-diglucoside. Furthermore, delphinidin-3-glucoside and cyanidin-3-glucoside were present too, as described in a previous study [21]. Major copigments of pomegranate are the tannins. The main copigment is punicalin with a [M-H]^+^ molecular ion at *m/z* 781. In addition, three isomeric punicalagins (*m/z* 1083) were detected (peaks C14, C16 and C20).

The red grape extract contained a lot of polymeric compounds, which were successfully separated by membrane chromatography. In the red grape anthocyanin fraction malvidin-3-glucoside [M-H]^+^ molecular ion at *m/z* 493 predominated. Furthermore, four anthocyanins were detected (delphinidin-3-glucoside *m/z 465*, cyanidin-3-glucoside *m/z 449*, petunidin-3-glucoside *m/z* 479, and peonidin-3-glucoside *m/z 463*) [22]. The phenolic composition of the copigment fraction of the red grape was dominated by flavonols. Isorhamnetin derivatives and quercetin derivatives were detected. Quercetin-3-glucuronide [M-H]^+^ molecular ion at *m/z* 477 is the main copigment in the examined *Vitis vinifera* red grape extract. Furthermore, gallic acid *m/z* 169 and caftaric acid *m/z* 311 were detected.

#### 3.1.2. Polymer Precipitation

**Figure 2:**
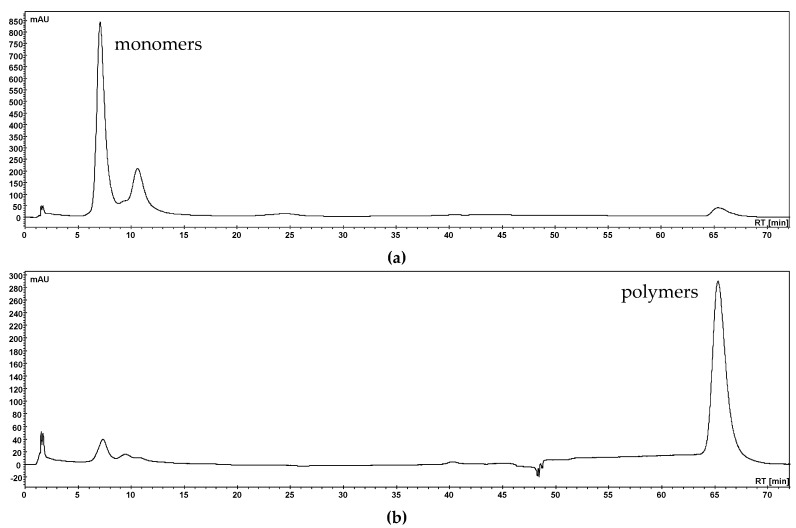
HPLC-PDA chromatograms after polymer precipitation with hexane at a wavelength of λ 280 nm of aronia NFC filtrate (**a**) and precipitate (**b**).

The polymer fractions (PF) of aronia NFC, pomegranate NFC and red grape JC extracts were obtained by hexane precipitation [9]. Monitoring of the precipitation reaction was performed by HPLC-PDA using a SIELC Primesep column [23]. As an example, the UV chromatograms of the aronia filtrate and precipitate are shown in Figure 2. Only polymeric compounds were detected in the precipitate fraction of aronia NFC whereas small amounts of polymers remained in the filtrate. The precipitation reaction was also successful with pomegranate NFC and red grape JC extracts.

#### 3.1.3. In Vitro Inhibition Study of Anthocyanin, Copigment, and Polymer Fractions of Aronia NFC, Pomegranate NFC and Red Grape JC

**Figure 3:**
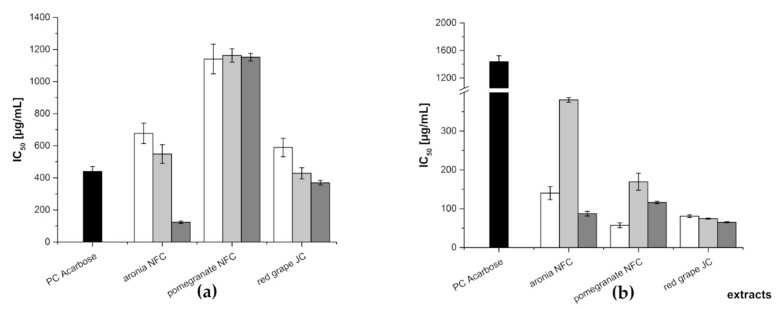
Inhibitory activities (IC_50_ values in µg/mL) of the three fractions (anthocyanin—white; copigment—gray; and polymer—dark gray) of the different juice extracts in comparison to the positive control (PC) acarbose for α-amylase assay (**a**) and α-glucosidase assay (**b**). Results are presented as means ± SD (*n* = 3).

The obtained fractions were investigated using two in vitro assays where the inhibition of α-amylase and α-glucosidase activity was measured. Acarbose, a pseudotetrasaccharide, which is a well-known α-amylase and α-glucosidase inhibitor, was used as the positive control (PC) in both assays. Its IC_50_ value was determined as 440 ± 30 µg/mL and 1439 ± 85 µg/mL for the α-amylase assay and α-glucosidase assay, respectively. Figure 3 shows the mean IC_50_ values for the anthocyanin, copigment, and polymer fractions of the different juice extracts. In the α-amylase assay, the polymer fraction of the aronia NFC extract was the most active amongst the tested samples exhibiting an IC_50_ value of 123 ± 8 µg/mL. The two other fractions of aronia showed a quite comparable inhibition potential (IC_50_: AF = 677 ± 63 µg/mL; CF = 548 ± 58 µg/mL). The three fractions of the pomegranate NFC extract were the less active ones amongst all tested samples (IC_50_: 1141 ± 93 (AF), 1163 ± 42 (CF), and 1152 ± 24 µg/mL (PF), respectively). A similar tendency as observed for the fractions of the aronia extract was determined for the red grape JC extract fractions. The IC_50_ values were 589 ± 57 µg/mL (AF), 428 ± 35 µg/mL (CF), and 369 ± 14 µg/mL (PF), in a comparable range of inhibitory activity.

In the α-glucosidase assay, all of the obtained fractions showed a higher inhibitory potential than the positive control acarbose. The less active one was the copigment fraction of aronia NFC extract with an IC_50_ value of 380 ± 6 µg/mL, followed by CF of pomegranate NFC extract with an IC_50_ value of 169 ± 22 µg/mL. The anthocyanin fraction of aronia NFC extract (IC_50_ = 140 ± 17 µg/mL) and the polymer fraction of pomegranate NFC extract (IC50 = 116 ± 3 µg/mL) showed a comparable inhibition level. All other fractions revealed nearly the same inhibitory potential against α-glucosidase, i.e., PF aronia NFC (IC_50_ values of 87.2 ± 5.9 µg/mL), AF red grape JC (80.9 ± 3.6 µg/mL), CF red grape JC (74.5 ± 1.4 µg/mL), PF red grape JC (65.0 ± 1.4 µg/mL), and AF pomegranate NFC (57.2 ± 6.4 µg/mL), respectively. Table 2 lists the IC_50_ values determined for all fractions in both enzyme inhibition assays.

### 3.2. Subfractionation with Countercurrent Chromatography and In Vitro Inhibition Study

The most active fractions in the two inhibition tests were the copigment and anthocyanin fractions of red grape JC and aronia NFC, respectively. For the identification of the key inhibitory compounds in these fractions, a further fractionation by high-performance countercurrent chromatography (HPCCC) was used [24]. HPCCC is a gentle and rapid liquid–liquid chromatographic technique for the preparative separation of natural compounds including phenolics. The separation with HPCCC allows us to prove the inhibition potential of individual constituents in the fractions by isolating them in sufficient amounts required for the inhibitory assays. In case of HPCCC, the separation of 1 g of a polyphenolic extract with a suitable biphasic solvent system was achieved within 1 h.

#### 3.2.1. Subfractionation of Anthocyanin and Copigment Fraction of Red Grape JC Using HPCCC

**Figure 4:**
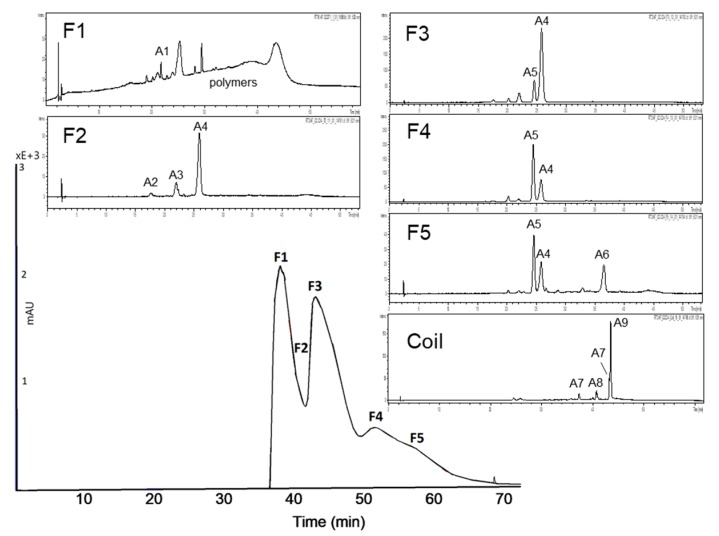
High-performance countercurrent chromatography (HPCCC) chromatogram at a wavelength of λ 520 nm of the anthocyanin fraction of the red grape juice concentrate (JC) sample and HPLC-ESI-MS chromatograms indicating the composition of each fraction.

The biphasic solvent system *tert-*butyl methyl ether/*n*-butanol/acetonitrile/water (2:2:1:5; v/v/v/v) acidified with trifluoracetic acid was applied for the separation of the anthocyanin fraction of red grape JC. The measured distribution coefficients of 0.5–1.9 predicted a good separation as presented in Figure 4. In the first fraction, polymeric compounds were concentrated und separated from the anthocyanins. Malvidin-3-glucoside, the main anthocyanin in red grapes, was detected in F2, F3, F4, and F5 due to co-elution with other anthocyanins. In F4 and F5 peonidin-3-glucoside was concentrated. Furthermore, four coumaroyl derivatives of anthocyanins were detectable in the coil fraction.

**Figure 5:**
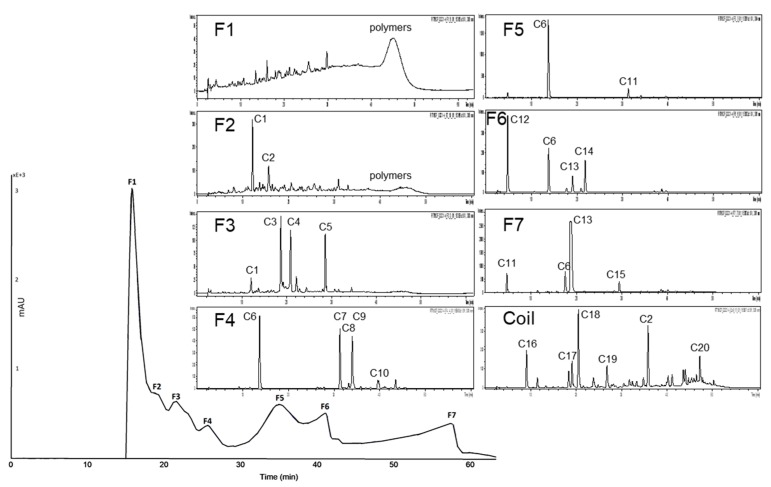
HPCCC chromatogram at a wavelength of λ 320 nm of the copigment fraction of the red grape JC sample and HPLC-ESI-MS/MS chromatograms indicating the composition of each fraction.

HPCCC fractionation of the complex copigment fraction of the red grape JC extract (RG-CP) was achieved with the biphasic solvent system *tert-*butyl methyl ether/acetonitrile/water (2:2:3; v/v/v). As presented in Figure 5 eight fractions were obtained. Also in the first fraction, polymeric compounds were concentrated und separated from the copigments. In F2 a coumaroyl derivative was concentrated together with an unknown compound. Moreover, in F5 caftaric acid was obtained in a purity of 88% by HPLC λ 280 nm. Minor compounds like resveratrol and syringic acid were concentrated in the coil fraction.

**Figure 6:**
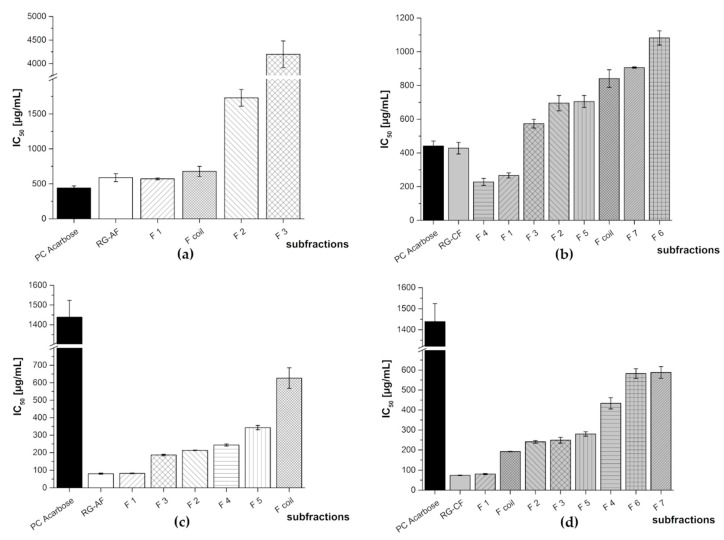
Inhibitory activities (IC_50_ values) of the HPCCC subfractions of red grape JC anthocyanin fraction (RG-AF) and red grape JC copigment fraction (RG-CF) in comparison to the positive control (PC) acarbose. Results are presented as means ± SD (*n* = 3). (**a**) RG-AF α-amylase assay (F4, F5: N/A), (**b**) RG-CF α-amylase assay, (**c**) RG-AF α-glucosidase assay, and (**d**) RG-CF α-glucosidase assay. N/A = not available.

Also these subfractions were submitted to biological screening which revealed that the subfractions of RG-AF and RG-CF had stronger inhibition activity against α-glucosidase than the positive control acarbose. In the case of the α-amylase assay, only RG-CF F4 and F1 exhibited a lower IC_50_ value than the positive control (see Figure 6).

RG-AF F1 was the most active subfraction in both assays. This activity is most likely due to residual polymeric phenols (tannins) which are present in this fraction. RG-AF F2, which consists of a mixture of malvidin-3-glucoside and petunidin-3-glucoside, exhibited a very potent inhibition of both enzymes. In case of RG-AF F4, consisting of malvidin-3-glucoside and peonidin-3-glucoside, and F5, containing additionally malvidin-3-acetylglucoside, no inhibition of α-amylase was observed whereas minor activity in the α-glucosidase assay could be detected.

In case of the copigment fraction of red grape (RG-CF), both inhibition studies identified F3, as active fraction which consists of a hexoside of coumaric acid and isorhamnetin. For the coil fraction (Fcoil), i.e., the fraction that remains on the HPCCC instrument after the separation has been completed), a substantial activity in the α-glucosidase assay could be measured. This fraction contains different copigments including coumaric acid, syringic acid, protocatechuic acid, epicatechin, and quercetin. Fractions 6 and 7 of RG-CF exhibited in both assays the lowest inhibitory activity. Both contained caftaric acid, gallic acid, and coutaric acid, while F6 additionally included fertaric acid and F7 resveratrol. All IC_50_ values of the different subfractions are listed in Table 3.

#### 3.2.2. Subfractionation of the Anthocyanin and Copigment Fraction of Aronia NFC Using HPCCC

**Figure 7:**
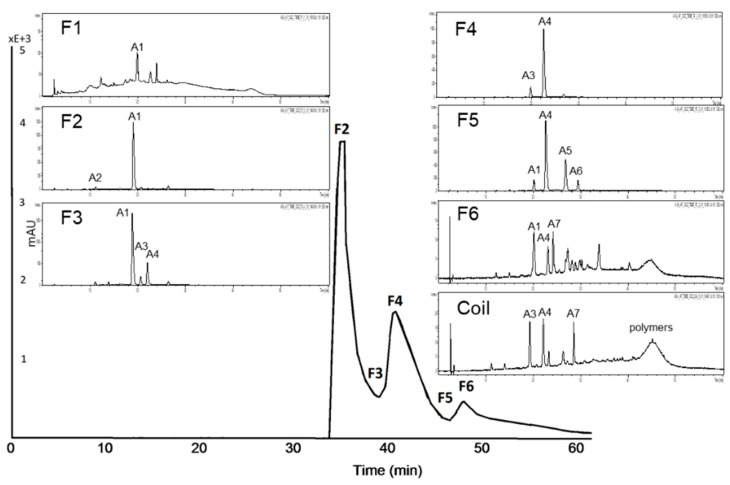
HPCCC chromatogram at a wavelength of λ 520 nm of A-AF and HPLC-ESI-MS/MS chromatograms of the composition of each fraction.

For the HPCCC separation of the anthocyanin fraction of aronia NFC a solvent system of *tert-*butyl methyl ether/*n*-butanol/acetonitrile/water (2:2:1:5; v/v/v/v) acidified with trifluoracetic acid was used. The chromatogram at a wavelength of λ 520 nm is presented in Figure 7. Each subfraction was analyzed with HPLC-ESI-MS/MS. Seven fractions, including the coil fraction, were obtained. Cyanidin-3-galactoside, the main anthocyanin of *Aronia melanocarpa*, was enriched in F1 and it could be isolated from F2 at a purity of 95% by HPLC λ 280 nm. In F4 cyanidin-3-arabinoside was concentrated in a purity of 87% by HPLC λ 280 nm. Polymers and two minor compounds (cyanidin derivative and an unknown compound) were detected after HPCCC subfractionation in the coil fraction. 

**Figure 8:**
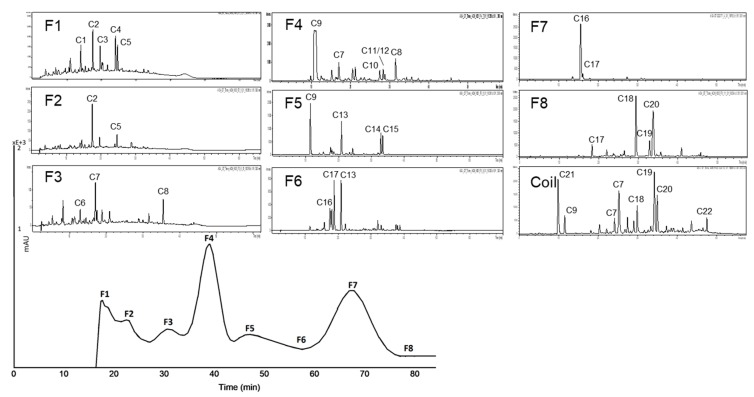
HPCCC chromatogram at a wavelength of λ 320 nm of A-CF and HPLC-ESI-MS/MS chromatograms of the composition of each fraction.

HPCCC fractionation of the complex copigment fraction of the aronia NFC extract (A-CP) was achieved with the solvent system *tert-*butyl methyl ether/*n*-butanol/acetonitrile/water (2:2:1:5; v/v/v/v), head to tail modus was used (cf. Figure 8). The measured distribution coefficients of 0.5–2.5 predicted a good separation. Ten minor compounds were detected after HPCCC subfractionation, which were mainly concentrated in the coil fraction. Chlorogenic acid (5-CQA), main copigment of A-CF, is enriched in F7. The other subfractions are still complex. Quercetin derivatives could be separated with HPCCC, due to their different sugar moieties (cf. F5, F8 and F coil).

**Figure 9:**
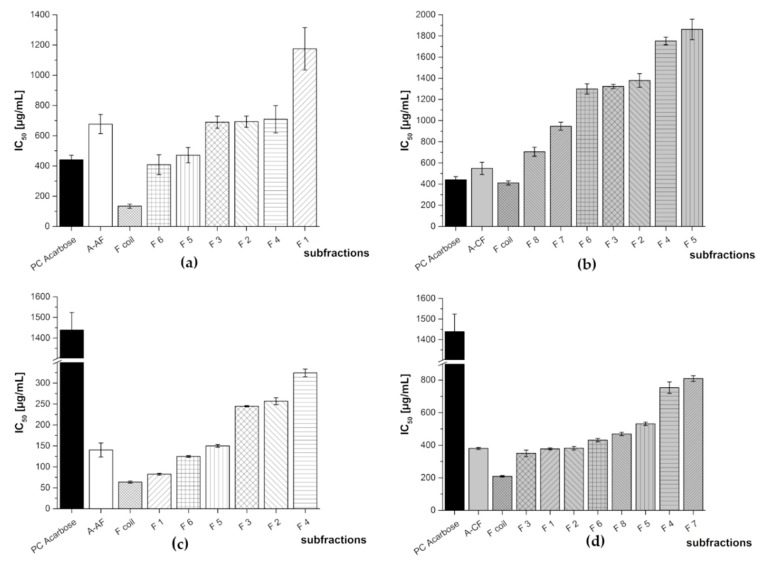
Inhibitory activities (IC_50_ values) of the HPCCC subfractions of aronia NFC anthocyanin fraction (A-AF) and aronia NFC copigment fraction (A-CF) in comparison to the positive control (PC) acarbose. Results are presented as means ± SD (*n* = 3). (**a**): A-AF α-amylase assay; (**b**): A-CF α-amylase assay (F1: N/A); (**c**): A-AF α-glucosidase assay; (**d**): A-CF α-glucosidase assay. N/A = not available.

In Figure 9 and Table 4 the results of the testing of A-AF and A-CF with the two inhibition assays are presented. There are clear differences between the tested fractions as well as the assays applied. With regard to α-amylase inhibition, the anthocyanin fraction of aronia (A-AF) was only moderately active, revealing highest activity for the coil fraction (F coil) as well as F6 and F5. The activity of F coil and F6 might be influenced by the presence of remaining polymeric phenolic compounds (tannins) whereas in the case of F5 the activity is clearly due to the presence of anthocyanin derivatives, such as cyanidin-3-arabinoside and cyanidin-3-xyloside. Also fractions F2, F3, and F4 are free of polymeric substances and the recorded activity can be clearly linked to the presence of monomeric anthocyanins (e.g., cyanidin-3-galactoside, cyanidin-3-arabinoside) in these fractions.

Contrary to the moderately active anthocyanin subfractions obtained from A-AF, all of the separated subfractions with copigments were considerably more active than the positive control acarbose. Composition of these fractions is depicted in Figure 8. 

Our results clearly indicated that α-glucosidase was very sensitive to anthocyanins. All of the separated subfractions were by far more active than the positive control acarbose. Interestingly with the copigment fractions lower inhibition rates are recorded which let us to conclude that for α-glucosidase inhibition mainly anthocyanins are responsible whereas for inhibition of α-amylase the copigments of aronia are more important.

#### 3.2.3. Subfractionation of the Anthocyanin and Copigment Fractions of Pomegranate NFC Using HPCCC

**Figure 10:**
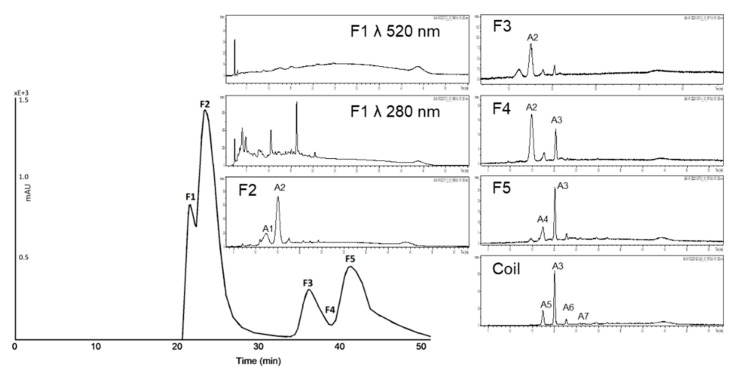
HPCCC chromatogram of AF of pomegranate NFC at a wavelength of λ 520 nm of P-AF and HPLC-ESI-MS/MS chromatograms of the composition of each fraction.

For subfractionation of the anthocyanin fraction of pomegranate NFC the solvent system *tert-*butyl methyl ether/*n*-butanol/acetonitrile/water (1:3:1:5; v/v/v/v) acidified with 0.1% TFA was used. Six fractions including the coil fractions were obtained. Figure 10 shows the UV chromatogram of the HPCCC separation at a wavelength of λ 520 nm and the HPLC-ESI-MS/MS composition of each fraction. In the first fraction traces of hydrolyzable tannins were detected as can be seen from the chromatogram recorded at λ 280 nm. All other fraction contained anthocyanins with cyanidin-3,5-diglucoside and cyanidin-3-glucoside being the major constituents. Furthermore, three minor contituents were concentrated in the coil fraction (delphinidin-3-glucoside, pelargonidin-glucoside and cyanidin-pentoside) in accordance with previous paper [21].

**Figure 11:**
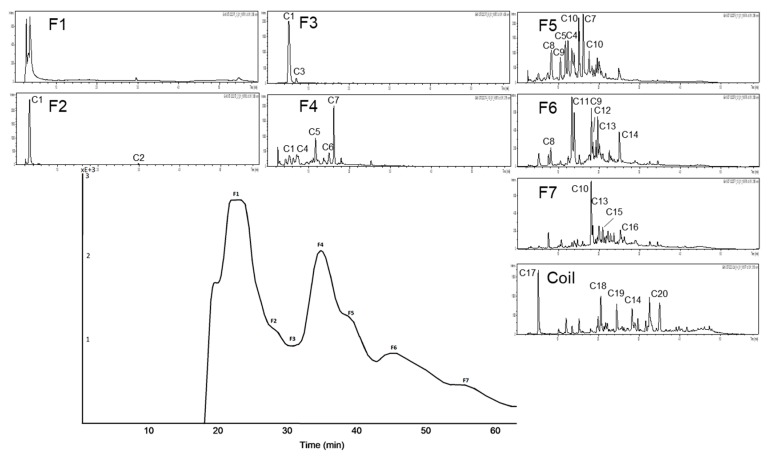
HPCCC chromatogram of CF of pomegranate NFC at a wavelength of λ 320 nm of P-CF and HPLC-ESI-MS/MS chromatograms of the composition of each fraction. Abbreviation: HHDP, hexahydroxydiphenyl.

HPCCC fractionation of the copigment fraction of pomegranate NFC was achieved with the solvent system *tert-*butyl methyl ether/*n*-butanol/acetonitrile/water (2:2:1:5; v/v/v/v) acidified with trifluoroacetic acid (Figure 11). Punicalin I, one of the major phenolic constituents of pomegranate NFC, could be isolated from F2 in a purity of 93% by HPLC λ 280 nm. The other subfractions are still complex and mainly contain different hydrolyzable tannins such as, e.g., punicalagin II and III [21]. Ten minor compounds were detected after HPCCC subfractionation, which were mainly concentrated in the coil fraction.

**Figure 12:**
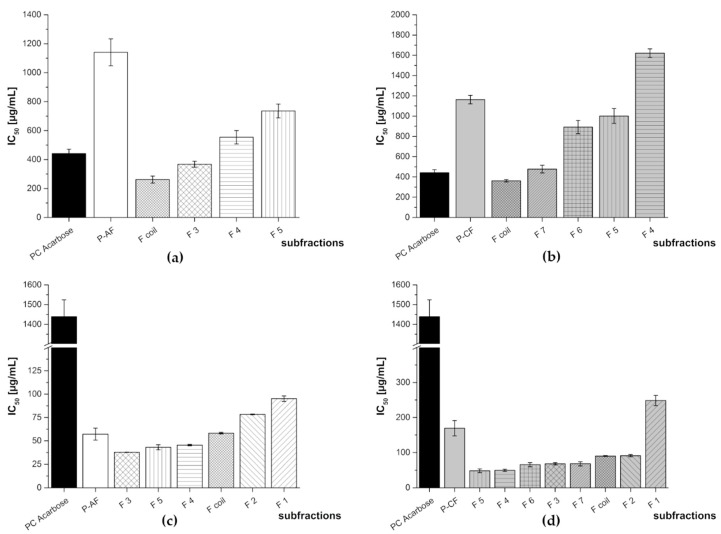
Inhibitory activities (IC_50_ values in µg/mL) of the subfractions of pomegranate NFC anthocyanin fraction (P-AF) and pomegranate NFC copigment fraction (P-CF) in comparison to the positive control (PC) acarbose. Results are presented as means ± SD (*n* = 3). (**a**) P-AF α-amylase assay (F1, F2: N/A), (**b**) P-CF α-amylase assay (F1, F2, F3: N/A), (**c**) P-AF α-glucosidase assay, and (**d**) P-CF α-glucosidase assay. N/A = not available.

The results for the enzyme inhibition studies of P-AF and P-CF are shown in Figure 12. The inhibitory potential of the subfractions was in all cases considerably higher against α-glucosidase compared to the positive control acarbose. In the α-amylase assay, the results were similar as obtained for the red grape JC and aronia NFC extracts. Again, some of the anthocyanin containing fractions exhibited an activity in the range of the positive control acarbose (F3 and F coil), while others (F4 and F5) showed a lower activity. P-AF F3, consisting of cyanidin-3,5-diglucoside, demonstrated the highest inhibitory activity in the α-glucosidase assay. Another comparative potential inhibitor of the digestive enzymes was F coil, containing cyanidin-3-glucoside, delphinidin-3-glucoside, pelargonidin-3-glucoside, as well as a cyanidin-3-pentoside.

Considering the results of the testing of P-CF in the α-amylase assay, no inhibitory activity could be determined for the punicalin containing subfractions F2, F3, and F4. Only fraction 7 and the coil fraction exhibited activities in the range of the positive control acarbose. F7 consisted of the tannins pedunculagin and punigluconin and in the coil fraction (F coil) a mixture of gallic acid and an ellagic acid derivative was present.

Higher inhibitory activities could be determined in the α-glucosidase assays. All of the subfractions showed an approximately ten-fold higher inhibition compared to the positive control acarbose which demonstrates that also hydrolyzable tannins are efficient inhibitors of α-glucosidase. Table 5 lists the IC_50_ values of the HPCCC subfractions of the pomegranate anthocyanin (P-AF) and copigment fractions (P-CF).

## 4. Discussion

For the identification of fruit juice constituents which inhibit the enzymes α-glucosidase and α-amylase an activity-guided fractionation of extracts from aronia (NFC), pomegranate (NFC), and red grape (JC) has been performed. Each of these extracts was separated in an anthocyanin and a copigment fraction using membrane chromatography. Additionally, by addition of hexane, the polymeric compounds were precipitated (polymer fraction). Hence, from each of the fruit juice samples a total of three phenolic fractions were obtained, i.e., the anthocyanin, copigment and polymer fraction, which were tested with regard to their potential to inhibit the digestive enzymes α-glucosidase and α-amylase in vitro. The most active fractions in the two inhibition assays were the polymeric fractions of aronia and red grape, respectively. These fractions consist of tannins, i.e., condensed flavan-3-ols, which are well-known protein precipitating agents [25,26]. The copigment and anthocyanin fractions of red grape also showed a high inhibitory potential against the two enzymes under study, in this case more specific interactions could be expected.

For an identification of the key inhibitors in these fractions, a further separation with HPCCC was carried out. HPCCC enables enrichment of individual compounds or mixtures of compounds in separated fractions which again can be tested with regard to their α-glucosidase and α-amylase inhibition potential in vitro. HPCCC subfractions containing peonidin-3-glucoside exhibited a lower inhibition potential against α-glucosidase and α-amylase, whereas delphinidin-3-glucoside and petunidin-3-glucoside containing subfractions were more active. This is in line with literature data which postulated that the structure of the anthocyanins, especially a high amount of hydroxyl groups and therefore polarity, plays an important role for the inhibition [3,6]. The same tendency holds true for the copigments. Phenolic substances rich in hydroxyl groups such as quercetin, pedunculagin, or punigluconin, showed a high potential to inhibit the activity of the two investigated digestive enzymes. Interestingly, subfractions containing punicalin showed no inhibitory effects in the α-amylase assay and only slight inhibition in the α-glucosidase assay. Obviously, low molecular phenolic compound are able to conduct more specific interactions with the enzymes and the inhibitory activity is not only related to the number of hydroxyl groups present in the molecule [27]. Furthermore, synergistic effects should also be considered as observed for cyanidin-3-galactoside. This anthocyan alone showed only a negligible potential to inhibit the two digestive enzymes while together in a mixture with cyanidin-3-arabinoside and cyanidin-3-xyloside it was very efficient. 

It has previously been reported that anthocyanins mainly inhibit the activity of α-glucosidase [28]. Our results are consistent with these findings and we could additionally demonstrate that copigments likewise inhibit α-glucosidase activity more potent than α-amylase-activity. Strictly speaking the fractions and subfractions were all even more active in the α-glucosidase assay than the positive control acarbose [29]. Our study also shows that it is not a single compound that is responsible for the inhibition of α-amylase and α-glucosidase activity. Whereas for α-amylase mainly the condensed tannins are efficient inhibitors, α-glucosidase is more susceptible to phenolic compounds and it could be shown that fruit-dependent major inhibition is also due to the copigments, e.g., in red grapes, or hydrolyzable tannins in the case of pomegranate. 

Taken together, we were able to identify active compounds using an activity-guided fractionation. But the use of an in vitro model of gastric glucose metabolism can only reveal some preliminary data. Some active ingredients from red fruit juice have been identified. But it has to be mentioned, that such in vitro results are first insights into structure–activity relationships, but these findings have to be evaluated with further studies preferably in vivo—e.g., performing human intervention studies measuring the blood glucose levels with and without these extracts—in the future.

## Figures and Tables

**Table:** 

**Peak**	**Compound**	**Peak**	**Compound**
A1	delphinidin-3-glucoside	A6	malvidin-3-acetylglucoside
A2	cyanidin-3-glucoside	A7	peonidin-3-coumaroylhexoside I+II
A3	petunidin-3-glucoside	A8	petunidin-3-coumaroylhexoside
A4	malvidin-3-glucoside	A9	malvidin-3-coumaroylhexoside
A5	peonidin-3-glucoside		

**Table:** 

**Peak**	**Compound**	**Peak**	**Compound**	**Peak**	**Compound**
C1	coumaroyl derivative	C8	quercetin-3-galactoside	C15	unknown
C2	unknown	C9	quercetin-3-glucoside	C16	protocatechuic acid
C3	coumaroyl-hexoside I	C10	quercetin derivative	C17	resveratrol
C4	coumaroyl-hexoside II	C11	quercetin-3-glucuronide	C18	coumaric acid
C5	isorhamnetin-3-hexoside	C12	gallic acid	C19	syringic acid
C6	caftaric acid	C13	coutaric acid	C20	epicatechin
C7	resveratrol-3-glucoside (piceid)	C14	fertaric acid		

**Table:** 

**Peak**	**Compound**	**Peak**	**Compound**
A1	cyanidin-3-galactoside	A5	cyanidin-3-xyloside
A2	cyanidin derivative	A6	cyanidin derivative
A3	cyanidin-3-glucoside	A7	unknown
A4	cyanidin-3-arabinoside		

**Table:** 

**Peak**	**Compound**	**Peak**	**Compound**	**Peak**	**Compound**
C1	dicaffeoylquinic ester I	C9	neochlorogenic acid (3-CQA)	C17	cryptochlorogenic acid (4-CQA)
C2	dicaffeoylquinic ester II	C10	coumaroyl quinic ester	C18	feruloylquinic acid II
C3	quercetin derivative	C11	quercetin-dihexoside I	C19	quercetin-pentoside
C4	caffeic acid-derivative	C12	quercetin-dihexoside II	C20	quercetin-hexoside
C5	unknown	C13	feruloylquinic acid I	C21	protocatechuic acid
C6	eriodictoyl-7-glucoronide	C14	quercetin-3-robinobioside	C22	quercetin
C7	coumaroyl derivative I + II	C15	quercetin-3-rutinoside		
C8	quercetin-3-vivianoside	C16	chlorogenic acid (5-CQA)		

**Table:** 

**Peak**	**Compound**	**Peak**	**Compound**
A1	delphindin-3,5-diglucoside	A5	delphinidin-3-glucoside
A2	cyanidin-3,5-diglucoside	A6	pelargonidin-glucoside
A3	cyanidin-3-glucoside	A7	cyanidin-pentoside
A4	pelargonidin-3,5-diglucoside		

**Table:** 

**Peak**	**Compound**	**Peak**	**Compound**	**Peak**	**Compound**
C1	punicalin	C8	digalloyl-hexoside	C15	caffeic acid hexoside
C2	ellagic acid	C9	granatin B I	C16	galloyl-HHDP-hexoside-pentoside
C3	punicalagin I	C10	pedunculagin I+II	C17	gallic acid
C4	ellagic acid derivatives	C11	granatin B II+III	C18	brevifolin carboxylic acid
C5	punicalagin II	C12	casuarinin	C19	valoneic acid bilactone
C6	galloyl-HHDP-hexoside	C13	punigluconin	C20	ellagic acid deoxyhexoside
C7	punicalagin III	C14	ellagic acid hexoside		

**Table 1:** HPLC-ESI-MS/MS data of separated anthocyanin and copigment fractions after membrane chromatography of aronia NFC, pomegranate NFC, and red grape JC.

Peak	[M-H]^+^	Fragments (*m/z*)	Compound	Peak	[M-H]^−^	Fragments (*m/z*)	Compound
A1	737	575, 287	cyanidin derivative	C1	153	109	protocatechuic acid
A2	707	575, 287	cyanidin derivative	C2	353	191, 179,135	neochlorogenic acid (3-CQA)
A3	449	287	cyanidin-3-galactoside	C3	353	191, 179, 161	chlorogenic acid (5-CQA)
A4	449	287	cyanidin-3-glucoside	C4	353	191, 179, 173, 135	cryptochlorogenic acid (4-CQA)
A5	419	287	cyanidin-3-arabinoside	C5	625	301	quercetin dihexoside I
A6	419	287	cyanidin-3-xyloside	C6	625	301	quercetin dihexoside II
A7	627	465, 303	delphinidin-3,5-diglucoside	C7	367	191, 179, 135	feruloylquinic acid
A8	611	449, 287	cyanidin-3,5-diglucoside	C8	595	301	quercetin-3-vivianoside
A9	465	303	delphinidin-3-glucoside	C9	609	301	quercetin-3-robinobioside
A10	479	317	petunidin-3-glucoside	C10	609	301	quercetin-3-rutinoside
A11	463	301	peonidin-3-glucoside	C11	463	301	quercetin-3-galactoside
A12	493	331	malvidin-3-glucoside	C12	463	301	quercetin-3-glucoside
				C13	781	601, 271	punicalin
				C14	1083	601, 299, 271	punicalagin I
				C15	783	601, 301	pedunculagin I
				C16	1083	601, 299, 271	punicalagin II
				C17	783	601, 301	pedunculagin II
				C18	951	907, 301	granatin B
				C19	783	601, 301	pedunculagin III
				C20	1083	601, 301	punicalagin III
				C21	799	479, 301	ellagic acid-derivatives
				C22	463	301, 257, 163	ellagic acid-hexoside
				C23	301	258	ellagic acid
				C24	169	125	gallic acid
				C25	311	179, 149	caftaric acid
				C26	325	175, 163	coutaric acid-hexoside
				C27	295	163	isorhamnetin-3-glucoside
				C28	477	316	quercetin-hexoside
				C29	477	301	quercetin-glucuronide
				C30	317	179	isorhamnetin
				C31	301	-	quercetin
				C32	285	151	kaempferol

**Table 2:** Inhibitory activities (IC_50_ values) of the three fractions (AF, CF, and PF) of the different juice extracts. Results are presented as means ± SD (*n* = 3).

Extract	Fraction	IC_50_ [µg/mL]	
		α-Amylase Assay	α-Glucosidase Assay
**PC Acarbose**		440 ± 30	1439 + 85
**aronia NFC**	AF	677 ± 63	140 ± 17
	CF	548 ± 58	380 ± 6
	PF	123 ± 8	87.2 ± 5.9
**pomegranate NFC**	AF	1141 ± 93	57.2 ± 6.4
	CF	1163 ± 42	169 ± 22
	PF	1152 ± 24	116 ± 3
**red grape JC**	AF	589 ± 57	80.9 ± 3.6
	CF	428 ± 35	74.5 ± 1.4
	PF	369 ± 14	65.0 ± 1.4

**Table 3:** Inhibitory activities (IC_50_ values) of the HPCCC subfractions of red grape JC anthocyanin (RG-AF) and copigment fraction (RG-CF). Results are presented as means ± SD (*n* = 3). N/A = not available.

Red Grape JC Fraction	Subfraction	IC_50_ [µg/mL]	
		α-Amylase Assay	α-Glucosidase Assay
**PC Acarbose**		440 ± 30	1439 + 85
**RG-AF**	F 1	571 ± 14	82.8 ± 1.9
	F 2	1730 ± 120	213 ± 1
	F 3	4198 ± 283	187 ± 5
	F 4	N/A	244 ± 6
	F 5	N/A	343 ± 12
	F coil	677 ± 72	626 ± 59
**RG-CF**	F 1	266 ± 15	80.7 ± 2.8
	F 2	695 ± 45	241 ± 7
	F 3	574 ± 26	249 ± 14
	F 4	228 ± 20	433 ± 28
	F 5	705 ± 36	280 ± 11
	F6	1082 ± 42	582 ± 25
	F 7	907 ± 4	588 ± 29
	F coil	841 ± 52	192 ± 1

**Table 4:** Inhibitory activities (IC_50_ values) of the HPCCC subfractions of aronia NFC anthocyanin (A-AF) and aronia NFC copigment fraction (A-CF). Results are presented as means ± SD (*n* = 3). N/A = not available.

Aronia NFC Fraction	Subfraction	IC_50_ [µg/mL]	
		α-Amylase Assay	α-Glucosidase Assay
**PC Acarbose**		440 ± 30	1439 + 85
**A-AF**	F 1	1175 ± 140	82.4 ± 2.2
	F 2	693 ± 37	257 ± 8
	F 3	690 ± 40	245 ± 1
	F 4	709 ± 91	324 ± 9
	F 5	471 ± 50	150 ± 3
	F 6	408 ± 66	125 ± 2
	F coil	134 ± 14	63.7 ± 2.4
**A-CF**	F 1	N/A	377 ± 6
	F 2	1379 ± 64	382 ± 11
	F 3	1323 ± 20	350 ± 19
	F 4	1753 ± 36	753 ± 35
	F 5	1862 ± 96	531 ± 10
	F6	1299 ± 49	431 ± 10
	F 7	947 ± 38	809 ± 17
	F 8	706 ± 42	469 ± 11
	F coil	411 ± 20	209 ± 5

**Table 5:** Inhibitory activities (IC_50_ values) of the subfractions of pomegranate NFC anthocyanin fraction (P-AF) and pomegranate NFC copigment fraction (P-CF) Results are presented as means ± SD (*n* = 3). N/A = not available.

Pomegranate NFC Fraction	Subfraction	IC_50_ [µg/mL]	
		α-Amylase Assay	α-Glucosidase Assay
**PC Acarbose**		440 ± 30	1439 + 85
**P-AF**	F1	N/A	95.2 ± 2.9
	F2	N/A	78.3 ± 0.4
	F3	367 ± 21	37.8 ± 0.2
	F4	554 ± 46	45.4 ± 0.8
	F5	736 ± 48	43.1 ± 2.7
	Fcoil	262 ± 24	58.2 ± 0.8
**P-CF**	F1	N/A	249 ± 15
	F2	N/A	90.9 ± 3.2
	F3	N/A	67.7 ± 3.1
	F4	1621 ± 42	48.5 ± 5.4
	F5	1001 ± 74	43.1 ± 2.7
	F6	892 ± 66	65.4 ± 5.7
	F7	477 ± 39	68.1 ± 5.7
	Fcoil	362 ± 12	90.1 ± 1.2
